# Editorial overview: The evolution of language as a neurobiological system

**DOI:** 10.1016/j.cobeha.2018.06.002

**Published:** 2018-06

**Authors:** Christopher I Petkov, William D. Marslen-Wilson

**Affiliations:** 1 Institute of Neuroscience, Newcastle University Medical School, Newcastle upon Tyne, UK; 2 Centre for Behaviour and Evolution, Newcastle University, Newcastle upon Tyne, UK; 1 Centre for Speech, Language and the Brain, Department of Psychology, University of Cambridge, Cambridge CB2 3EB, UK

**Current Opinion in Behavioral Sciences** 2018, **21**:v–xii

For a complete overview see the Issue

Available online 27th June 2018

**https://doi.org/10.1016/j.cobeha.2018.06.002**

2352-1546/© 2018 The Authors. Published by Elsevier Ltd. This is an open access article under the CC BY license (http://creativecommons.org/licenses/by/4.0/).

“*By no effort of the understanding, by no stretch of the imagination, can I explain to myself how language could have grown out of anything which animals possess, even if we granted them millions of years for that purpose ... Language is our Rubicon, and no brute will dare to cross it*” — Max Müller, 1887

“*We have learned that language evolution keeps repeating itself; the same is bound to occur to theories about language evolution.*” — Willem Levelt, this issue

Neuroscience and medicine in the 21st century face important challenges that depend on better understanding how human cognition and neurobiology share fundamental properties with other species and the important ways in which they differ. A salient problem in this context is to understand the neurobiological and evolutionary context in which human language emerged — a key enabling capacity that drove our remarkable success as a species. An explanatory account of this process will need to be continually informed by emerging insights into common principles of brain organisation and evidence for key points of divergence across the species.

This special issue brings together both leaders and upcoming bright stars in their respective scientific fields, who have been asked to reflect on the challenging question of how we should approach the origins of human language, viewed as a neurobiological system. The 31 articles making up the special issue consider, from a remarkable diversity of perspectives, a broad set of themes contrasting the apparent uniqueness of language and its properties in the modern human with a wide range of evidence for direct (and indirect) neurobiological precursors in our primate relatives and ancestors, and for analogous capacities in broader cross-species comparisons. Topics such as domain-general and domain-specific aspects of language and evidence for evolutionary conservation and specialisation recur throughout the issue, combined with contributions on the general principles of behavioural, cognitive and neural systems and their relevance for understanding language as a complex neurocognitive system.

We encouraged all contributors to stray from their comfort zones and to consider perspectives outside of their immediate field of study, including diverging views within and beyond this collection. In this way, the interdisciplinary discourse between linguists, comparative behaviourists and language neuroscientists can be influenced by scientists who do not work on language or language evolution, and vice versa. In so doing, we hope that our readers will find many sources of insight and inspiration distributed through these pages.

*Section one sets the stage with perspectives on the uniqueness of language and its genetic fingerprint*. Willem Levelt’s masterly historical piece empowers us to look ahead as we glance back, further than usual. As Levelt says, it is a ‘sobering experience’ to be reminded that the basic set of questions about language evolution have hardly changed since scientific thinking about the human condition started to emerge in the mid-18th Century. His article makes us uncomfortably aware of how little explanatory progress has actually been made since then. Levelt takes us on a witty and erudite tour of the ‘sleeping beauties’ of the field — theories of language evolution that re-emerge under different guises — including the recent reincarnation of ‘miracle theories’ of language as a divinely or otherwise suddenly endowed human ability. In the next piece, James Hurford, distilling a lifetime of combat with the problem of language evolution, strips apart two prominent but diametrically opposed theories of how human language emerged. The resulting conclusions reveal the illuminating consequences of breaking down these theories into their core postulates.

Dan Dediu and Stephen Levinson reassess perspectives on the antiquity of human language following a flurry of new genetic, paleontological and archeological data. They consider whether language evolution was gradual or saltatory and the extent of differences between Neanderthals and modern humans, which they argue to be substantially less than previously thought. They view Neanderthals as ‘fully articulate beings’ with advanced linguistic capacities, in support of a strongly gradualist account of the emergence of human and human-like language and communication.

In his stimulating and combative piece W. Tecumseh Fitch takes the evolutionary picture further back, in order to position a novel two component theory of sequential linguistic structure building in the modern human, and to relate these theories to evolutionarily conserved capacities detectable in nonhuman animal species (e.g. ten Cate and Kikuchi *et al.*, this issue). His ‘phonological continuity hypothesis’ posits that these evolutionary *finite state* combinatorial capacities underpin human combinatorial phonology — the organisation of phonemes into complex sequences. The second part of his approach — the ‘dendrophilia hypothesis’ — is that hierarchical phrasal syntax (the building of ‘trees’) requires a higher-level *supra-regular* grammar. This is a capacity for which he sees no convincing evidence in any nonhuman species. Its emergence in the human requires the inherited finite state sequencing capacities to be augmented by the attachment of a memory ‘stack’ achieved in Broca’s area — most likely by virtue of its links to parieto-temporal storage areas. This renders Broca’s area a key locus for the human capacity for hierarchical syntax (see also papers by Friederici, Rouault & Koechlin, Flinker & Knight).

The perspective by Bart de Boer and Willem Zudeima resonates with Fitch. The authors discuss the evolution of combinatorial structure in language, focusing on the evidence base and theories on combinatorial phonology. They also conclude that the prerequisites for combinatorial capacities are conserved, even though uniquely human cultural pressure required vast vocabularies and open-ended means to syntactically structure meaningful expressions.

Kenny Smith pinpoints a key property of human language — that it exploits the combinatorial structure of signals to convey complex meanings — and asks how this *compositionality of language* could have evolved. Iterative language evolution studies in his laboratory indicate that the emergence of compositionality in humans crucially depends on the need to communicate with others. Taking a cultural evolutionary perspective, Smith highlights the growing evidence that the rudiments of the capacities necessary to underpin the emergence of compositionality are present in nonhuman animals. Nick Chater and Morten Christiansen come to similar conclusions, but from a different perspective, viewing the acquisition of language purely as skill learning, no different from the acquisition of any complex cognitive skill, and not requiring a ‘universal grammar’ or the gradual expression of such during a child’s first years. They too emphasise cultural over biological factors in determining the properties of human language and argue that domain general capacities for chunking and rule-based structure building are as important for language and its acquisition as they are for sensorimotor and cognitive function.

The section concludes with two perspectives from evolutionary genetics. Nicky Staes, Brenda Bradley, William Hopkins and Chet Sherwood seek to enrich the picture of the genetic roots of language by examining genes associated with enhanced social and communicative skills — factors undoubtedly critical to the emergence of human language. Taking FOXP2 as one well-studied example, the authors illustrate its importance in underpinning aspects of verbal communication by relating differences in vocal output across primate species to variations in FOXP2 expression. They go on to emphasise the importance of much less well studied genes involved in the regulation of social behaviour, that also vary across humans and apes, including those linked to social communication, prosociality and cooperation.

Martin Kuhlwilm reviews the latest gene network analyses from ancestral human DNA, focusing on the coding regions that lie next to or interact with the FOXP2 gene. The recent work identifies gene ‘deserts’ or deletions after different ‘admixture events’ where archaic hominin lineages (Neanderthal and Denisovan) interbred with the ancestors to modern humans. A striking feature of the modern human genome, subsequent to these events, is the apparent removal of the resulting genomic changes from particular regions. One of the largest of such deserts is found in the region around FOXP2, suggesting an incompatibility between humans and their archaic relatives in this region. This underlines, through an unexpected route, the importance of FOXP2 and surrounding coding regions in the emergence of the modern human (compare with perspective by Dediu and Levinson).

*Section two focuses on comparative animal behaviour and on language development in children*. Taking the capacity for vocal learning as a critical aspect of human language, and lamenting the narrow range of animal models currently studied — passerine birds being the dominant species — Ella Lattenkamp and Sonja Vernes emphasise the need for a broader cross-species approach. They point to recent observations of capacities for vocal learning in several understudied non-primate mammalian species, and call for a well-founded set of structured comparisons to place human vocal learning capacities in a richer and better understood evolutionary context.

Focusing on birds, Carel ten Cate reviews current research using artificial grammar learning to extract insights into convergent capacities, in birds relative to humans, for structured sequence (rule-based) learning. While numerous experiments on different species of birds reliably show the capacity to learn abstract patterns of adjacent and nonadjacent dependencies between the training items, methodological questions remain about the basis for these discriminations — in particular whether they reflect unintended lower-level regularities. On ten Cate’s view, the jury is still out where the extent of grammar learning by birds is concerned. Michael Griesser, David Wheatcroft and Toshitaka Suzuki take a quite different approach to structured sequence interpretation and production in birds, focusing on evidence for compositional syntax in the natural call combinations observed in two species of birds. While this is far from complex hierarchical human syntax, the authors argue that these combination calls have the key property of communicating a compositional meaning by virtue of the meaning of their components. The authors go on to analyse the evolutionary pressures under which this capacity arises, and propose a research programme for determining whether these combined calls are indeed semantically compositional (see Smith and Züberbuhler articles in this issue).

Shifting to nonhuman primates, Robert Seyfarth and Dorothy Cheney highlight the emerging evidence for the pragmatic flexibility of primate vocalisations used to facilitate social interactions and reduce uncertainty about social intentions. Detailed analyses in primate species — chiefly in baboons and bonobos — of the modulation of vocalisations by the social context of utterance, are overturning the classical dichotomy between learned flexible vocalisations in humans and the fixed repertoire of innate and invariant vocalisations attributed to nonhuman primate species (see also the piece by Hage). This theme resonates with a number of other contributions in this issue: Asif Ghazanfar and Diana Liao review the evidence for developmental flexibility in vocal sound production in marmosets, a highly social and vocal species who, like humans, are cooperative breeders where an extended family assists in raising the young. They propose that in marmosets, as in humans, parental influences interact with physical changes to transform an infant’s vocalisations into their mature form (also see Lattenkamp & Vernes article in this issue).

Klaus Zuberbühler considers the nature of primate combinatorial capacities, providing a thorough and critical analysis of a wide range of comparative work on artificial grammar learning and natural vocal production. Zuberbühler notes the substantial evidence for various types of signal combination in primate vocalisations, but leaves open as outstanding questions whether any of the reported combinations count as compositional, and whether and which forms of animal combinatoriality were an evolutionary substrate for human syntax.

Taking a statistical learning perspective, Alice Milne, Ben Wilson and Morten Christiansen provide a detailed and insightful analysis of structured sequence learning across sensory modalities in human and nonhuman primates, with the goal of determining how far abstract domain-general systems can be identified in both groups, potentially relevant to language function in the human and its evolutionary underpinnings. Where humans are concerned, they conclude that there is unlikely to be a unitary cross-modal sequence processing mechanism, with separable systems engaged by auditory and visual input systems. Preliminary behavioural and neuroimaging evidence from nonhuman primates suggests similar conclusions, but Milne and colleagues stress that these are very early days in probing these complex cross-species comparisons, undoubtedly of great promise for understanding the domain-general substrate for key aspects of human language function.

Turning to child development, Judit Gervain proposes an intriguing view of language development that emphasises the role of the infant’s prenatal experience, as an important aspect of the human adaptation to the challenge of language acquisition faced by every new member of the species. Although acoustic signals are highly filtered by the womb, rhythmic and other types of structured patterns appear to predispose the developing perceptual system of a child, providing a ‘prenatal prosodic bootstrapping’ for subsequent language acquisition. This knowledge of the prosodic patterns of their native language not only helps the neonate to discriminate their mother tongue from other environmental sounds but also to parse the speech stream into linguistically relevant prosodically marked units, thereby guiding early recognition of the lexical and syntactic structure of the language.

Jutta Mueller, Alice Milne and Claudia Maennel underscore the importance of understanding how children learn non-adjacent sequencing dependencies as a scaffold for their language learning in the first year. They review comparative EEG work in human adults and infants and monkeys using artificial grammars that emulate non-adjacent dependencies. This research reveals intriguing differences and parallels between these three groups, with human infants outperforming adults in implicit learning tasks, and showing largely comparable EEG signals to nonhuman primates. These ontogenetic and phylogenetic comparisons provide important insights into a potential evolutionarily conserved ability to automatically extract non-adjacent relations from auditory sequences.

*Section three considers some fundamental properties of neural systems as constraints on the emergence of language as a neurobiological system*.

Marion Rouault and Etienne Koechlin remind us that not only language, but also the complexity of our cognitive function in general distinguishes us from other animals. They take on the challenge of proposing how language function can be integrated with theory on prefrontal cortical (PFC) function — a region which also plays a salient role in conventional accounts of language. Rouault and Koechlin view PFC as a complex system of inferential and hierarchical control processes that compute the optimal adaptive solution for guiding behaviour in an uncertain, changing and open-ended world. At the algorithmic level, this account has several aspects relevant for language, in particular in the domain of hierarchically organised behaviour, which the authors see as applied both at the linguistic discourse level and at the level of sentence-level tree-structures.

Taking a different tack, Josef Rauschecker asks where language came from given that the evolutionary process appears to have been frugal in exapting neural mechanisms shared with nonhuman primates. He proposes that the link across the species is that the primate brain is fundamentally designed to infer internal models via different processing streams: ventral fronto-temporal pathways are crucial for sensory to meaning mapping and dorsal pathways for sequential analysis are required for syntax in humans. The core building blocks and operations, Rauschecker notes, are evolutionarily conserved and engage neural processing pathways, including those for vocal production, that have differentiated in humans in ways that still need elucidation (also see Mars *et al.*, Hage and Friederici in this issue).

Using microstructural histological analyses, assessing the cyto- and receptor-achitecture of Broca’s region and surrounding prefrontal cortical areas, Karl Zilles and Katrin Amunts provide a masterful overview of the neuroanatomical structure of the key Brodmann areas 44 and 45, superseding the classical maps provided a century ago by Brodmann and by von Economo. Any research attempting to elucidate the functions of these structures will need to take on board the additional segregation of these areas into multiple subregions and their extensive integration into adjacent frontal areas. Zilles and Amunts not only comment extensively on the relationship between the microstructure of Broca’s region and brain function, but they also provide a novel and informative cross-species comparison between human Broca’s region and the arguably homologous regions in our primate relatives, from the macaque to the chimpanzee. Despite the wide differences in brain size between these species, Zilles and Amunts conclude that these cortical areas are surprisingly similar in cytoarchitecture and connectivity, given the apparently major qualitative differences in their respective functional roles.

Henry Evrard considers the Von Economo and fork cell neurons — specialised cell-types that were initially thought to be unique to humans and to have a special role in the evolutionary emergence of human intuition and awareness, as well as being implicated in a range of neuropsychiatric disorders and fronto-temporal dementias. Evrard and colleagues, following earlier discoveries that these neurons also occurred in large-brained species such as elephants and dolphins, showed that they were also found in the macaque brain — specifically, as in humans, in the anterior insula. Evrard summarises the current wide-ranging experimental investigations of the role of these neurons in interoception and cognition and proposes a neurobiologically specific model of the primate anterior insula, territory just ventral to inferior frontal regions often associated with language functions in humans.

Patrick McNamara and Raymon Durso conclude this section with an intriguing overview of two major dopaminergic networks — the frontal-parietal network (FPN) and the ‘social brain’ network– each with characteristic roles important for cognitive and language functioning. The FPN network, heavily involving prefrontal cortex (see also Rouault & Koechlin piece) is argued to be critical for syntactic processes, while the social brain network mediates higher order pragmatic processes, relating language outputs to their social context of use. Focusing on Parkinson’s disease — primarily a progressive depletion of dopaminergic cells in the brain — McNamara and Durso argue that the pattern and timing of language processing deficits as the disease progresses in these patients help to elucidate the different but complementary roles played by the two major dopaminergic networks and their role in language.

On a sombre note, the neuroscience community was greatly saddened by the untimely death of Howard Eichenbaum in July 2017. When we approached Howard at the planning stage for this special issue, he was enthusiastic about taking on the challenge of describing the neuroscientific principles underpinning the hierarchical organisation of space and time in the hippocampal memory system and of exploring the potential relevance of these for language. Although we cannot know what Howard would have written, we can speculate that his ground-breaking work demonstrating that time cells and place cells in the hippocampus encode temporal and spatial relationships between stimuli could well be extended to the challenge of evolving neural systems that establish and maintain parallel dependencies within and between words and phrases in language. Neural principles of ‘relational memory’ generated from the study of memory systems could well play a key role in understanding the evolutionary emergence of complex human language.

*The final section discusses neural systems for speech and language alongside evidence for neural conservation and human specialisation.*

The first three papers in this section consider the core neural specialisations in humans for language. Angela Friederici takes a nativist position, arguing that only humans possess a biologically predetermined system of rules and operations that permit the combination of words into hierarchically structured phrases and sentences. This capacity, furthermore, reduces to a basic computational mechanism (`Merge’) that binds elements into a hierarchical structure. Friederici focuses first on recent evidence from her own research programme pointing to a location in posterior BA 44 as the critical cortical region supporting Merge. She then turns to the ontogenetic and phylogenetic context for the major dorsal fibre tracts connecting BA 44 to posterior temporal brain regions also critically involved in human language processing. Friederici notes that some aspects of sequence processing and their neurobiological substrates are now known to be shared with nonhuman primates (see also Milne *et al.*, Mueller *et al.*, and Kikuchi *et al.* papers in this issue). In humans, the dorsal arcuate fasciculus interconnecting area 44 with temporal cortex develops in mid to late childhood to support language proficiency and uniquely distinguishes us as a species (also see perspectives by Rauschecker and Mars *et al.*, this volume), together with the evolutionary emergence of the Merge function itself.

In counterpoint, Peter Hagoort is sceptical that much hinges on whether language-related neural activity is found in area 44 or 45, or even whether cognitive or language function relates meaningfully at all to notions of Brodmann-style brain areas. He argues that a detailed understanding of the neurobiology of language is needed at multiple functional rather than structural levels, from the local properties of canonical microcircuits in neocortex to the large-scale networks supporting language function across the human brain (see also Mars *et al*. paper this issue). Hagoort makes the point, furthermore, that until we do have mechanistic computational accounts of this multi-level processing architecture, the evolutionary stance is going to be of limited value — in effect, that we need to know *what* evolved before we can profitably speculate about *how* it evolved.

Distinct from the Hagoort and the Friederici approaches, William Marslen-Wilson and Mirjana Bozic propose an approach to language evolution that places the neurobiological and evolutionary origins of language centre stage. This Dual Neurobiological Systems approach sees the communicative and combinatorial capacities of the modern human as reflecting a dynamic coalition between two interacting but evolutionarily and functionally distinguishable systems: A left lateralised fronto-temporal system, with potentially human-unique properties, is crucial for complex morphosyntax, while a bilateral lexically and semantically oriented system, with characteristics largely inherited from our primate ancestors, supports broader capacities for social cognition and pragmatic interpretation (see also Staes *et al.* paper in this issue). The authors conclude by focusing on the notion of a neurobiologically defined *ancestral state* — asking where in the human lineage can we locate the crucial transition from the communicative capacities provided by the ancestral bihemispheric primate system to the modern human with the enhanced capacities provided by the emergence of the left hemisphere system.

In relation to the classical notion of Broca’s area as crucial for structured speech production, Adeen Flinker and Robert Knight consider evidence from recent human intracranial recording (Electrocorticography; ECoG) studies, which reveal in remarkable spatiotemporal detail the dynamics of neural activity as words are heard and then produced. Neural activity in Broca’s area precedes speech output by around 250 ms, possibly reflecting a conserved functional role also visible in marmoset monkey inferior frontal neurons at similar delays before vocalization onset. More broadly, Flinker and Knight address the open question of what cognitive processes Broca’s area supports (see also Rouault & Koechlin and McNamara & Durso). Where speech production is concerned several recent ECoG studies of high gamma dynamics suggest that Broca’s area has a lead role integrating information across cortical regions in parallel and sequencing an articulatory code for motor cortex implementation. Where the processing of hierarchical structure is concerned — often thought to be a key function of these inferior frontal regions (e.g. Friederici, this issue) — they conclude that while current neuroimaging studies do provide evidence for frontal involvement in processing linguistic structure, as part of a more distributed fronto-temporo-parietal network, it remains to be seen what unique role Broca’s area plays within these networks.

Karen Campbell and Lorraine Tyler confront the fundamental question of what is domain-general and what is domain-specific in human language function — a critical issue for any evolutionary account, where domain-general processes are candidates for evolutionarily conserved capacities, while domain-specific processes are *prima facie* candidates for human-unique specialisations. Focusing on spoken language comprehension, they use independent components analysis (ICA) to decompose the on-going fMRI signal into separable component networks. Comparing natural listening conditions with task-based conditions, these techniques allow a domain-specific syntax system to be differentiated both from the wider language system (including domain-general components responsible for semantics and pragmatics) and from broader domain-general networks (regulating, for example, attention and memory) which come on-line to support performance on specific tasks, but are not intrinsically required for syntax and involve quite distinct neural systems.

The final three papers of the special issue consider correspondences and differences between human and nonhuman primate neurobiology. Rogier Mars, Nicole Eichert, Saad Jbabdi, Lennart Verhagen and Matthew Rushworth report on an important research programme that seeks to determine the ‘common blueprint’ shared by all primate brains, viewed in terms of the architecture of connections between brain areas. This provides a principled empirical cross-species basis for determining what is conserved and what is novel specialisation in cases such as human language. Mars and colleagues summarise here the outcome of this on-going programme for two domains — the longitudinal pathways (dorsal and ventral) connecting frontal regions to other brain areas, and the neural mechanisms underlying vocal learning and control. In both cases, the cross-species comparative method, though not always straightforward to put into practice, motivates a set of testable hypotheses on how the relevant pathways may have differentiated in humans.

Yukiko Kikuchi, William Sedley, Timothy Griffiths and Christopher Petkov propose a relational knowledge hypothesis, where an ancestral neural system capable of establishing temporal dependencies is integrated with human language processes that depend on analogous operations in time. This hypothesis unifies cross-species behavioural and neuroimaging studies probing sequence learning, using established artificial grammar techniques, with concepts from the predictive coding framework and the results of recent studies mapping the oscillatory dynamics underpinning sequence processing. Neurophysiological data from monkeys and ECoG human data show striking similarities in cross-frequency coupling in humans and monkeys, providing specific evidence for evolutionarily conserved neural processes underpinning language-relevant computational functions, and consistent with a broader ‘relational knowledge’ account of the perception and learning of environmental regularities.

Steffen Hage rounds off the special issue by offering a new view of the origin of human vocal control capacities. Taking as a starting point the increasing wealth of evidence for cognitive flexibility in nonhuman primate vocal production (see related pieces by Seyfarth & Cheney and Ghazanfar & Liao), Hage argues that monkeys possess preadaptations that are crucial for the emergence of a learned vocal communication system such as human speech. On this basis he proposes a dual neural network model for the human brain that includes a volitional articulatory network originating in prefrontal cortex (BA 44 and 45) that cognitively controls the vocal output of a phylogenetically conserved primary vocal motor network that is common to all primate species (see Flinker & Knight, this issue). Critically, recent evidence from recording and stimulation studies with macaques suggest that the homologue areas to BA 44 and 45 in these species also participate in voluntary control of vocal output from the primary vocal motor network. As Hage concludes, this emerging link between prefrontal executive structures and the ancient vocal motor system is likely to be one of the key preadaptations in the primate lineage to speech acquisition in humans.

*In conclusion*, this striking collection of short essays, lively snapshots of dozens of different intensive research programmes, convey the impression of a vibrant field making strong progress — and largely, we believe, because the evolution of human language is indeed being placed in its neurobiological evolutionary context. It is from this perspective that we can lay to rest Max Muller’s defiant remarks from the distant past. We may not yet fully understand how `language could have grown out of anything which animals possess, even if we granted them millions of years for that purpose’, but we know, first, that there is one ‘brute’ that did manage this — our own species — and we are, second, finally coming to grips with the many deep and complex ways in which our heritage as intelligent, social primates has shaped our language-using capacities as modern humans.

Almost all the work in this special issue presupposes a gradualist approach to the evolution of human language, and generally avoids the ‘single factor’ type of approach that attempts to explain language evolution in terms of a single determinative evolutionary change. Instead we see a conception of language evolution as a multi-factorial process, with many different strands being pursued.

A key focus of interest is of course the sequential complexity of language as a signalling system, and many contributions in this collection focus on the evolutionary relationship between combinatorial sequence learning and analysis processes in nonhuman animals and the nature of sequencing in human language. It is clear that major recent progress has been made in this regard, and that combinatorial capacities in vocal production and learning are more broadly evolutionarily conserved than was thought, such that we can start to probe these commonalities across species at levels of analysis much closer to the neural mechanisms involved. Though we should bear in mind that these conserved capacities may be limited to finite-state levels of grammatical relationships, and that a gap still remains between the sequential dependencies that nonhuman species can learn and those necessary to represent and generate the hierarchical structural relationships that characterise human linguistic outputs.

A parallel issue, also critical for fully bridging the human-nonhuman gap where the sequential communication of meaning is concerned, is whether the structured strings generated by some nonhuman species can be said to be semantically compositional — a defining aspect of human language. There are intriguing glimpses of the rudiments of compositionality in the combination calls of two species of birds and in some primate species. But it is not clear at this point whether such rudiments are sufficiently widespread, especially in nonhuman primates, to be treated as evolutionary precursors to the compositional properties of human language. A relevant and related concern, that is not given full treatment anywhere in the special issue, is whether the vocal (or indeed gestural) outputs of any nonhuman species can be treated as ‘symbolic’ in nature. Beyond the well-known examples of the vervet monkey ‘leopard’ and ‘eagle’ alarm calls, how far can non-human vocalisations be said to be symbolic in nature — where this is assumed to be a pervasive and foundational property of human signalling sequences?

A second important set of strands concerns the long assumed gulf between modern humans and their last common primate ancestor in their capacities both for vocal learning and for voluntary control of their vocal outputs. While it remains clear that our closer primate relatives show impoverished capacities where vocal learning is concerned (in the sense of learning new sounds or combinations of sounds for communication purposes), a wealth of evidence has emerged — much of it touched on in this special issue — for cognitive control and modulation of primate vocalisations in their natural social contexts. Indeed, on the account proposed by Steffen Hage, links between prefrontal executive centres and vocal motor output can already be detected in the monkey brain, indicating evolutionarily conserved neural pathways highly relevant to the further developments in the human system. These are potentially major changes in the evolutionary background to the degree of cognitive control seen in the human system.

What also emerges from the articles in this collection is the surprising degree of apparent qualitative correspondence between neural substrates and structures in humans and in nonhuman primates where frontal and temporal language relevant neural systems are concerned. So much so that even for Broca’s region Zilles and Amunts were led to ask: “*But how could humans develop a complex language on the basis of neuroanatomical conditions principally not different from those of non-human primates?”* — though Mars and colleagues might well reply that this is the unsurprising consequence of the common blueprint underlying all of these primate brains. In any event, as our understanding of the levels of conservation improves so should potential clarity as to the specific form of human specialisations.

The whole, it seems, is greater than the sum of the evolutionarily conserved parts. To take a core example, a left lateralised fronto-temporal system involved in linguistic operations on a large semantic store develops in ontogeny as a result of cultural and developmental experiences while the child’s brain and abilities mature. How this system specialises and the extent to which it interfaces with evolutionarily conserved processes needs to be much better understood mechanistically and across neural scales. This requires further study of language-specific and cognitive domain-general processes in humans and an understanding of which cognitive domain-general processes are also evolutionarily conserved. To achieve this requires both comparative research with other animals and neuroimaging studies with humans that use analysis techniques better adapted to identifying the networks involved and to characterising their developmental trajectories.

Our interim conclusion, then, is that the scientific community is together making outstanding progress in this complex project — one that has been described as the most challenging problem in science. At the same time, inevitably, we are only at the beginning — and probably not even at the ‘end of the beginning’. So much still remains to be understood with regards to conserved capacities or divergences across species related to core aspects of language operations, their genetic bases and neural mechanisms. Yet, the path taken and the challenges surmounted so far in understanding language evolution should give us cause for celebration. We can be confident that a more complete understanding of the brain and of human neural specialisations, including but not exclusively for the emergence of language and communication, can only be achieved in a neurobiologically rooted evolutionary context.

*This special issue is dedicated to the memory of Professor Howard B. Eichenbaum, an exceptional neuroscientist whose curiosity about the neural instantiation of space and time will be a continuing inspiration*.

